# Frailty-Informed Decision Making During Acute Health Crises: A Competency-Based Framework for Person-Centered Care

**DOI:** 10.3390/jcm14248968

**Published:** 2025-12-18

**Authors:** Paige Moorhouse, Laurie H. Mallery

**Affiliations:** Centre for Health Care of the Elderly, Dalhousie University, 5595 Veterans’ Memorial Lane, Halifax, NS B3H 2E1, Canada; laurie.mallery@nshealth.ca

**Keywords:** frailty, advance care planning, goals-of-care discussions, shared decision making, dignity-conserving care, substitute decision making

## Abstract

Frailty profoundly influences the risks, benefits, and outcomes of medical care, yet clinical decision making during an acute health crisis is often driven by single-system treatment paradigms rather than a holistic understanding of patient values, prognosis, and personhood. This review outlines a practical, competency-based framework to guide clinicians through key clinical tasks that reliably align treatment decisions with what matters most to individuals living with frailty. The approach distinguishes between interventions that remain medically reasonable versus those that are unlikely to provide benefit and acknowledges that appropriate treatments evolve as frailty progresses. Anchoring the framework is an emphasis on dignity and the preservation of personhood through every stage of care. Much like a clinical procedure, these clinical tasks require defined intent, structured steps, and practice to ensure competence. When performed skillfully, these tasks may reduce low-value interventions, support autonomy, and promote compassionate, goal-concordant care during the most challenging moments of aging.

## 1. Introduction

Frailty is the baseline state of vulnerability due to the loss of reserve capacity that results from the accumulation of health and social stressors over the life course. Clinically, frailty presents as changes in cognition, mobility, and function that impact day-to-day life and independence. While multiple studies have described the concept of frailty and demonstrated its construct validity across different clinical contexts, few address the clinician’s complex and nuanced role in guiding patients and their circle of care through the trajectory of decline that characterizes the last chapter of life in frailty. Conceptual models emphasize situational diagnosis and shared decision making when caring for complex older adults (e.g., Amblàs-Novellas et al. [1].) and outline competencies for shared decision making in frailty and multimorbidity (e.g., van de Pol et al. [2]). This article extends these concepts by translating them into a practical, task-based framework that delineates the specific clinical actions required to operationalize frailty-informed decision making during acute health crises. When mastered, these tasks enable a dynamic, individualized approach to care that aims to maximize dignity and personhood and minimize low-value and harmful interventions. The following clinical vignette is used to demonstrate the application of each competency in sequence; however, this framework can be applied in an iterative way, as required by changes in health status, cognition, or the care context.

**Case:** Mr. L is an 85-year-old man with Alzheimer’s dementia, diabetes, and osteoarthritis that limits mobility. He lives at home with his wife and has been admitted to the hospital with chest pain and investigations consistent with a non–ST elevation myocardial infarction (NSTEMI). Coronary angiography reveals diffuse three-vessel coronary artery disease, including disease in the left main artery. The cardiology team is considering whether to proceed with coronary artery bypass grafting (CABG) (coronary anatomy is not amenable to Percutaneous Coronary Intervention) or to pursue medical management, prompting the need for a structured, frailty-informed decision-making process.

## 2. The Seven Task-Based Competencies

### 2.1. Task 1: Routinely Identify and Stage Frailty

Several tools for identifying frailty have shown construct and predictive validity, such as the Clinical Frailty Scale (CFS) [3], the Fried Phenotype [4], and the Frailty Index [5]. The CFS, an ordinal scale ranging from 1 (very fit) to 8 (very severely frail), classifies frailty based on functional, cognitive, and mobility characteristics. Like other frailty identification tools, the CFS demonstrates robust predictive validity for key outcomes such as mortality, hospitalization, and institutionalization [3,6,7]. In addition to its predictive validity, the CFS is well-suited to support decision making in frailty due to its widespread clinical use, ordinal classification that offers more granularity than non-frail, pre-frail and frail [4], its descriptive, language (as opposed to an index score) [5] and its conceptual alignment with Reisberg’s Functional Assessment Staging Tool (FAST) which describes dementia stages [8]. This concordance enables clinicians to relate the frailty level to dementia stage. For example, an individual with mild stage dementia would correspondingly be mildly frail on the CFS [3]. The CFS provides a practical framework to summarize a patient’s overall health status and inform care planning. The CFS also lends itself to integration with clinical information systems to help routinely identify individuals who would benefit from care planning and goals of care discussions.

Accurate staging of frailty using the CFS depends entirely on reliable information from appropriate sources and careful consideration of the factors that influence function and cognition [9], Individuals in both hospital and community settings commonly overestimate functional independence and mobility compared with observed performance [10,11]. When frailty is driven by dementia (or even Mild Cognitive Impairment) loss of insight is an early and characteristic feature [12,13]. For this reason, the functional history should be obtained from a collateral historian, someone who has directly observed the individual’s abilities or is familiar with how they manage instrumental activities of daily living (e.g., driving medication management, financial management, cooking, and cleaning) and basic activities of daily living (e.g., dressing, bathing, grooming, eating and toileting). The functional history should describe the extent and trajectory of functional dependence and specify whether changes are related to cognitive, physical, or combined limitations. Dementia is a common but often under-recognized driver of frailty that significantly impacts whether and how standard care interventions are likely to benefit patients [14]. Most frailty assessments do not include standard measures to assess cognition, and the CFS is no exception. Although the CFS accounts for the presence and severity of dementia in its scoring, it does not incorporate a standardized cognitive test to determine diagnosis or severity. Including a validated cognitive measure as part of routine frailty assessment can identify the need for more detailed evaluation, inform selection of a decision maker (see below), and clarify the frailty stage [9].

**Case application.** The clinician obtains collateral history to assess baseline frailty. Mr. L’s wife reports progressive cognitive deterioration over four years. For two years prior to his NSTEMI, Mr. L had been dependent on his wife for banking, shopping, cooking, cleaning, and medication administration. In the last six months. Mrs. L had noted that her husband required prompting to bathe at appropriate intervals and change his clothing. However, once cued, Mr. L retained the ability to carry out the steps of bathing and dressing independently. He ambulated mostly within the home using a cane and had sustained two falls in the past 6 months when transferring out of bed. Mrs. L indicates her husband’s cognition does not appear to be worse in hospital. Based on collateral history and bedside cognitive testing, Mr. L is diagnosed with moderate stage dementia. He is identified as moderately frail on the CFS. Together, these findings provide a baseline for communication and care planning.

### 2.2. Task 2: Translate Frailty into a Meaningful Clinical Construct for Patients and Substitute Decision Makers

**Figure 1:**
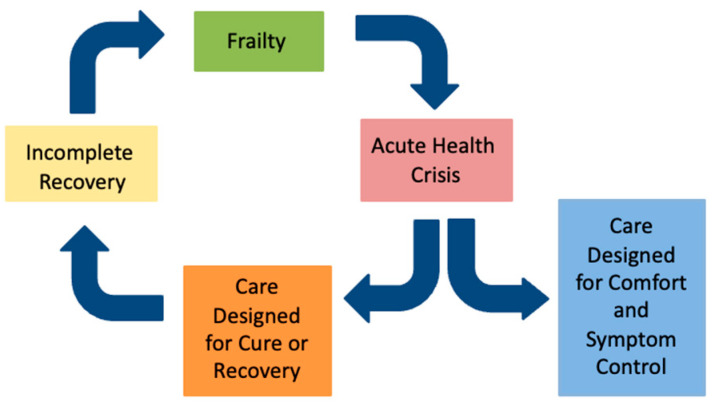
The frailty cycle.

The term “frailty” can be confusing, because its clinical definition can differ from how the word is used by the public. Health professionals use the term to describe a syndrome of vulnerability and risk that is distinct from normal aging [15], whereas the public may view frailty as a part of normal aging or even as a pejorative label [16,17,18,19,20]. Effectively communicating the concept and meaning of frailty requires clinicians to acknowledge these difference and frame frailty in a way that promotes understanding, dignity, and collaboration. The authors propose the Frailty Cycle (Figure 1) as a conceptual model that translates the abstract concept of frailty into a recognizable pattern of health vulnerability and a trajectory of decline commonly seen after health events and interventions [21].

**Figure 2:**
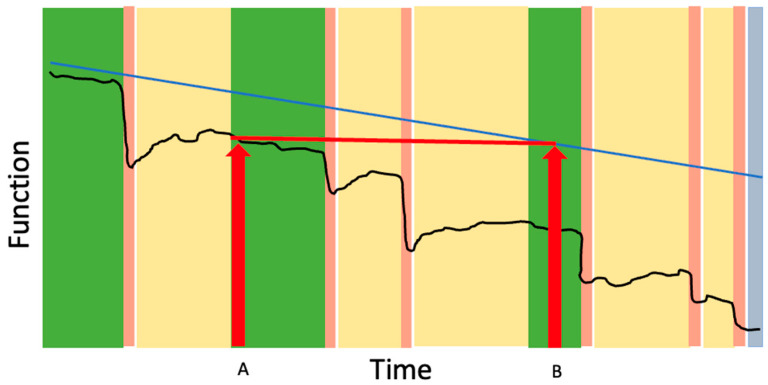
The impact of the frailty cycle on functional trajectory. The black line represents typical changes in function over time for an older adult living with frailty. Periods between crises (green bars) represent relative stability or gradual decline. Acute health crises (pink bars) trigger sudden drops in function. Although some recovery occurs when curative or rehabilitative therapies are applied (yellow bars), individuals often establish a new, lower functional baseline rather than returning to their prior level until end of life (blue bar). The blue line shows the hypothetical trajectory of function if acute crises did not occur, illustrating that losses seen after a crisis (Time A) reflect a level of function that otherwise might not have been reached until much later in the course of frailty (Time B). Across repeated crises, the cumulative effect is an acceleration of functional decline and progressive loss of independence.

In contrast to earlier work suggesting a slow, linear decline in function [22], the frailty trajectory more closely resembles that of end-stage organ failure [23], with one important distinction: as frailty progresses, recovery from acute illness (“the health crisis”) tend to become increasingly incomplete, leading to further frailty and vulnerability (Figure 2). The health crisis may result from an acute exacerbation of a chronic health issue (e.g., congestive heart failure); a new health issue (e.g., pneumonia), or an elective procedure (e.g., hip arthroplasty). Most people living with frailty will experience a stepwise decline in function and quality of life with each health crisis in the last months or years of life.

The Frailty Cycle provides several insights that can inform clinicians’ understanding of frailty and help frame expectations with patients and families:

- Recovery in frailty is often incomplete: Many patients and their caregivers have lived experience with incomplete recovery following the acute health crisis, such as a stepwise decline in cognition following delirium or a permanent need for a gait aid after surgery. Describing the Frailty Cycle can help validate what can otherwise be an isolating and mystifying experience of “he’s never been the same since” by helping to frame the cost of recovery as often involving permanent changes in cognition, mobility or function that may require adaptations and changes in care needs. This concept has important implications for provider education and patient communication including the insight that patients do not need to be back to their pre-hospital baseline in order to be discharged home; the key is ensuring safety and adequate supports based on their current level of function and needs.
- Health crises can accelerate the trajectory of decline: As shown in Figure 2, a health crisis in frailty can accelerate the trajectory of functional decline, such that the functional level reached after incomplete recovery (Time A) mirrors the level that would have occurred months or years later without the event (Time B). The acceleration of dementia progression after delirium is one example of this concept [24]. The concept is particularly useful for helping patients understand the risks of elective procedures. For example, a patient with moderate frailty who undergoes an elective open abdominal aortic aneurysm repair may experience a new, lower functional baseline after surgery—one that otherwise might not have been reached until much later in the trajectory had the stressor not occurred. This reality underscores that, for many frail patients, elective procedures intended to prolong life in the setting of asymptomatic disease can paradoxically pose an immediate threat to current quality of life and independence.
- End of life in frailty often looks different than expected: The Frailty Cycle frames frailty as a life stage-one that most people will experience if they live long enough. For many, the end of life with advanced frailty differs from the commonly expressed hope of “passing away peacefully in my sleep,” a contrast that can guide realistic and compassionate care planning.
- Frailty is unlikely to be eradicated or prevented entirely: The Frailty Cycle provides a meaningful translation of the stochastic and deficit accumulation models of aging [25]. The stochastic model describes aging as the cumulative effect of random cellular damage, while the deficit accumulation model explains how this damage manifests clinically as the gradual buildup of health deficits. Both models illustrate how biological systems lose the capacity for repair and compensation over time—a principle consistent with the second law of thermodynamics, which describes the inevitable drift toward disorder in complex systems. As health and social stressors accumulate, physiologic reserve and redundancy are depleted until repair becomes incomplete and damage accumulates. Eventually, even small stressors can precipitate failure. This principle underlies the frailty index (FI), which models both the rate of deficit accumulation and the threshold at which accumulated deficits become incompatible with survival [26]. In this way, clinical frailty can be seen as a byproduct of medical and societal advances that prolong life by allowing individuals to survive with chronic disease. These principles explain why frailty is increasingly prevalent and highlight opportunities to improve the experience of living with frailty by reducing the frequency of acute health crises and supporting personhood and dignity while acknowledging the immutability of frailty’s drivers.

**Case application.** The clinician interprets Mr. L’s current health and proposed interventions through the lens of the Frailty Cycle and considers how Mr. L’s moderate could affect recovery from CABG. Further progression of his moderate stage dementia would be expected even without surgery, and surgery represents a major risk for delirium, incomplete recovery, and stepwise deterioration in cognition and mobility. Framing CABG within this trajectory clarifies that successful revascularization would not halt dementia progression, and that surgery may accelerate decline in cognition, mobility, and independence.

### 2.3. Task 3: Identify the Decision Maker(s)

Informed decision making in frailty is complex. It requires decision makers to understand the patient’s health status and prognosis and appreciate how current and future treatments may affect their quality of life and function. Achieving this level of understanding depends on the clinician’s ability to provide clear information, interpretation, and guidance in the form of specific recommendations [14,27]. The decision maker is the patient (when they have capacity), and this responsibility is transferred to the Substitute Decision Maker (SDM) when the patient’s capacity is impaired. In addition to receiving appropriate information, a patient’s ability to participate in informed decision-making depends on having the cognitive capacity to understand, interpret, and apply information to their own health and values. The guidance offered here is intended as a general clinical framework recognizing that legal definitions of capacity and consent requirements vary across jurisdictions.

For older adults, particularly in settings such as inpatient medicine and long-term care, where frailty and cognitive impairment are highly prevalent, clinicians should adopt a more deliberate and nuanced approach to shared decision making. The common assumption that “patient-centered care” means exclusively patient-driven decision making must be reconsidered in the context of frailty, and the prevailing principle—that capacity should be assumed unless there is a reason to doubt it—also warrants scrutiny in this setting.

Estimates of cognitive impairment in acute medical wards suggest that a majority of older adults benefit from a closer evaluation of their capacity to provide consent and direct their care. In this setting, Mild Cognitive Impairment (MCI) affects approximately 62% of patients, dementia 2.3% [28], and delirium 30% [29]. In long term care, roughly 69% of residents have dementia, and a further 18% have undiagnosed dementia or other causes of cognitive impairment. Although MCI is defined by preserved function despite cognitive deficits, individuals with MCI demonstrate difficulty with some elements of informed consent related to complex decisions [30,31]. For these reasons, assessing capacity for complex decision making should be a standard step before all care planning discussions.

The assessment of capacity for complex decision making need not be onerous. Clinicians should limit their assessment to the decision(s) at hand and the patient’s capacity at the present time. Simple, open-ended questions can provide valuable insight—for example, “Tell me about the health issue that brought you into hospital.” “What treatments or tests are being considered?” and “How might these treatments help you, and what are some the risks?” It is critical to distinguish between a patient’s ability to assent to, or express a preference for a treatment, and their ability to provide fully informed consent. This distinction is especially important when there is cognitive impairment. A patient’s lack of insight into their diagnosis of dementia—a chronic, progressive, and terminal condition—significantly limits their capacity to make complex care decisions that will impact, and be impacted by, dementia. Beyond understanding the conventional risks and benefits of treatments, patients must also appreciate how dementia progresses over time; how it affects tolerance of, and recovery from interventions; and how complications such as delirium can accelerate decline [14]. Without this understanding, capacity for complex decision-making cannot be established.

In frailty, there are significant risks to assuming capacity. First, when interventions are presented as the standard of care, patients may interpret this as a recommendation and may passively assent to burdensome treatments without understanding the risks—especially for interventions that don’t require a formal consent process. Next, assuming capacity where it does not exist undermines autonomy because the selected choice may not represent the patient’s values or interests. Finally, incorrect assumption of capacity may squander an opportunity to engage a substitute decision maker.

Capacity in frailty exists on a continuum. The goal is to identify the optimal balance of shared decision making among the patient, their SDM, and the care team to maximize autonomy and ensure informed consent, as follows:

- When patients demonstrate full capacity, they should still be encouraged to include their SDM as a decisional understudy to bear witness to information and to the values guiding the patient’s approach to decision making.
- When capacity is partially compromised, the SDM should participate in developing a care plan based on the patient’s known preferences and prognosis, which can then be reviewed with the patient for input or awareness.
- Finally, when capacity is significantly compromised, the SDM and care team should work together to create and implement a plan consistent with the patient’s values and prognosis and help the patient engage with the plan wherever possible.

Effective, frailty-informed care planning requires thoughtful and courageous application of a patient’s values and priorities to guide treatment decisions. This may mean forgoing interventions intended to prolong life if the interventions would compromise quality of life, function, or dignity. An appropriate goal may be to square the curve of functional deterioration, by avoiding interventions that are expected to involve an extended period of disability and dependence.

Substitute decision makers also require substantial support. While they are not the direct recipients of treatment, they bear the emotional burden of the role and the consequences of the decisions they make on the patient’s behalf. Most SDMs are spouses or adult children, who assume the role by default and often feel unprepared [32]. This underscores the importance of involving SDMs early and consistently, even when patient capacity remains intact, so they can fully understand the patient’s values, goals, and health [33]. SDMs often experience moral distress and mental health morbidity related to their role, particularly from efforts to avoid or live with decisional regret about choices made [33,34]. For this reason, it is often appropriate to have the SDM involve their own support person during care planning conversations.

**Case application.** A conversation with Mr. L to assess his capacity for decision making indicates that he is. is unaware of the timeline and reasons for his current hospitalization. He cannot provide an account of his health issues, and he denies any concerns about his memory or functional independence. The clinician concludes that Mr. L lacks capacity to make complex medical decisions as dementia limits his ability to weigh long-term risks, appreciate the consequences of surgery on cognition and function, and appreciate how dementia progression may impact recovery. His wife is identified as the substitute decision maker. She participates attentively in discussions with health professionals, guided by her understanding of Mr. L.’s values.

### 2.4. Task 4: Achieve a Shared Understanding of Current and Future Health

Once the decision maker(s) has been identified, the next task is to provide them with a holistic understanding of the patient’s health and anticipated trajectory. A detailed approach to information gathering and sharing is described elsewhere [27,35] but should include the following:

1. **Understand the decision maker’s personal experience with frailty, dementia, and progressive health decline**: Health literacy refers to the degree to which individuals can obtain, process, and understand basic health information and the services needed to make appropriate health decisions [36]. Personal experiences with illness, caregiving, or loss strongly influence health literacy and can shape attitudes toward medical decisions [37,38]. Inviting the decision maker to reflect on these past experiences can provide powerful insights into regrets, worries, or trauma that may shape their attitudes, engagement, and preferences in current decisions. For example, witnessing prolonged or uncontrolled suffering before death may influence a decision maker’s priorities for early comfort-focused care. Conversely, a prior traumatic experience with dying, such as a medical error or iatrogenic complication, may make it difficult for the decision maker to trust or engage with the idea that this experience can be different.
  Exploring previous experiences with severe frailty or advanced illness also helps the provider frame information in a way that is relatable. For example, “Since you cared for your aunt with dementia, you’re likely familiar with how dementia progresses gradually, but often with a stepwise decline following hospitalization or delirium.” Directly soliciting the decision maker’s worries or fears about the future can serve as a useful starting point for helping them articulate their values and priorities (e.g., “I’m hearing you say that you’re worried about feeling short of breath at the end of life. If comfort is a priority, we can make sure medications are available to ease that sensation rather than waiting for an ambulance to arrive”).
2. **Share expectations about what future health might look like with and without the proposed procedure**: Applying medical evidence to frail older adults is particularly challenging. Frail older adults, by virtue of their age or comorbidities, are routinely excluded from large, randomized control trials, the source of most clinical practice guidelines [39,40,41]. As a result, clinicians often lack condition-specific data that meaningfully applies to this population. Prognostic tools such as ePrognosis [42] or the PiPS [43] score can be useful adjuncts when discussing expected outcomes, but they should be interpreted within the broader context of the individual’s frailty, trajectory, comorbidities, and recent health crises. In frailty, when there are multiple progressive conditions, life extension may not be the patient’s primary goal. In this context, the concept of the Frailty Cycle can be instrumental in helping the decision maker understand the risks of incomplete functional recovery and the possible impacts on comfort and dignity.
3. **Highlight the implications of multiple conflicting causes of mortality in frailty**: Most people who are frail have multiple chronic progressive health issues, which carry two key implications for decision making. First, treating or addressing current or future health conditions to prolong life often extends the period spent progressing through the stages of the remaining health conditions. For example, although treating aortic stenosis with transaortic valve replacement (TAVR) in the setting of moderate-stage dementia may relieve symptoms of aortic stenosis, it also removes aortic stenosis as a potential cause of death, leaving the patient to progress from moderate to advanced stages of dementia—an outcome that may not align with the patient’s values. Second, interventions aimed at prolonging life may lose benefit when other life-limiting conditions or competing causes of mortality are present. The provider’s role is to help the decision maker carefully choose interventions that are most likely to shape an end-of-life consistent with the patient’s values and priorities. For instance, for a patient with end stage renal disease, congestive heart failure, and an ischemic limb, it may be preferable to prioritize addressing dyspnea and ischemic pain, recognizing that an end-of-life trajectory related to renal disease may be preferable to the trajectory that would result from pursuing renal replacement therapy.

**Case application:** The clinician arranges a meeting with Mrs. L and recommends she invite others who may want to hear information about Mr. L. Mrs. L identifies their daughter as a key support and invites her to the meeting. During the meeting, the clinician introduces the diagnosis of dementia, including its current stage, expected progression, and prognosis, framing moderate stage dementia as a stage at which shifting the focus of care toward symptom management may be appropriate depending on the patient’s values. Mr. L’s wife describes his prior experience as a caregiver to his mother who developed dementia and lived in a long term care facility during the last three years of her life. The clinician reviews the current health crisis including the standard of care (CABG) and alternative options (medical management). The clinician introduces the concept of incomplete recovery in frailty after surgery (i.e., the Frailty Cycle) and what this might mean for Mr. L’s cognition and mobility. The clinicians also reviews that even without complications, CABG may add years to life that are spent progressing to more advanced stages of dementia. Finally, the clinician reviews that without surgery, Mr. L.’s left-main disease and multivessel disease carries a high risk of recurrent ischemia and mortality, and what symptom-based management of coronary artery disease is expected to involve including management at home and possible visits to the emergency department for symptom control.

### 2.5. Task 5: Attend to Dignity and Personhood

**Figure 3:**
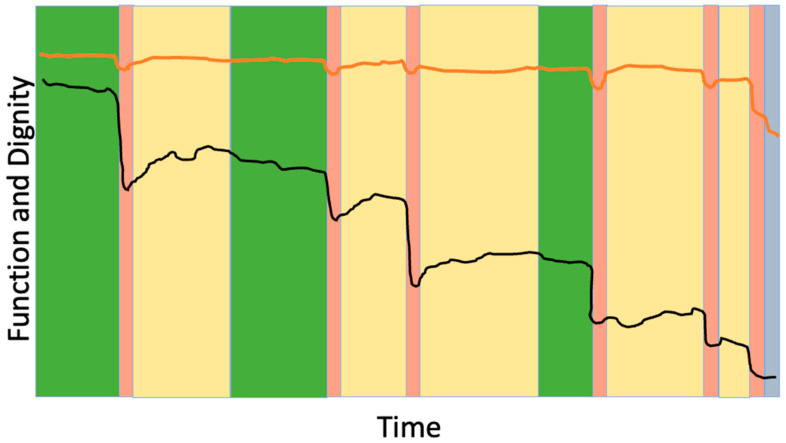
Preserving dignity as the goal of frailty-informed care. The black line represents changes in physical function over time for an older adult living with frailty, showing episodic declines after acute health crises. The orange line represents the individual’s self-perceived dignity or personhood. The goal in advanced frailty is to maintain dignity and sense of self even as function declines. Color coding as in Figure 2.

The suffering associated with loss of dignity may be more distressing than physical pain [44]. The term “personhood” refers to the recognition and preservation of an individual’s inherent identity, value, and roles—the aspects of self that make someone who they are—especially in the face of illness or vulnerability. The concept of fractured personhood—the feeling of, “I don’t feel like the person I used to be,” has been applied to end-of-life care but is also particularly relevant to frailty, which may be experienced as a prolonged disruption of identity and biography. While we may not be able to reverse the progression of frailty or the functional dependence that accompanies it, “squaring the trajectory of loss of dignity” is a feasible and meaningful aim (Figure 3). This concept refers to maintaining personhood and dignity until the very end of life, thereby minimizing the period of dignity erosion. The foundational first step in achieving this is understanding what dignity means to the patient.

Evidence-informed tools can help clinicians elicit and clarify personhood by asking the Patient Dignity Question, “What should I know about you as a person to help me take the best care of you that I can?” [45]. This question can also be asked of a SDM and can clarify how treatments may impact individual elements that underpin personhood. For example, for a person with a history of sexual or physical abuse, receiving hands on assistance with personal care may be a triggering experience. In approaching decision making for this person, providers and decision makers should consider whether life-prolonging therapies are likely to result in a new baseline level of function that would include the need for such assistance. The next steps in squaring the trajectory of loss of dignity involve planning ahead to align care with the patient’s values and prognosis and preparing to respond to future health crises in ways that preserve dignity and personhood.

Centering personhood and dignity in the context of frailty helps illuminate the forms of suffering inherent to an acute health crisis that may not otherwise be visible to decision makers. Hospitalization—particularly in emergency settings—commonly disrupts a person’s identity and sense of self through unfamiliar surroundings, absence of trusted caregivers and routines, reduced autonomy, and the confusion and distress associated with delirium [46]. These experiences represent a threat to personhood and can erode dignity, especially when hospitalization occupies a substantial proportion of the remaining lifespan [47]. Recognizing the likelihood and impact of this type of suffering in frail older adults provides decision makers with a more fulsome understanding of what recovery may entail, allowing them to more accurately apply the risks and benefits of proposed interventions to the patient’s values.

**Case application.** Mr. L’s wife says he identifies as an avid fly fisher and golfer. In more recent years he enjoys visits with his grandchildren and a weekly coffee group with two long-term friends. She is able to articulate his long-held values: remaining at home, recognizing family for as long as possible, spending time outdoors, and avoiding prolonged hospitalization. The clinician explores how surgery could threaten these aspects of personhood—particularly if cognitive decline accelerates or if Mr. L is no longer able to ambulate independently. They discuss how medical management, despite a higher risk of cardiac symptoms and reduced life expectancy, may better preserve dignity and personhood.

### 2.6. Task 6: Identify Which Treatment Decisions Should Be Decided in Advance

Advance care planning is a process that supports adults at any age or stage of health in understanding and sharing their personal values, life goals, and preferences for future medical care [48]. When health is complex, planning for future health crises can be challenging because the timing and nature of health events can be difficult to predict. In addition to establishing the decision maker’s understanding of the concept of the health crisis in frailty, and achieving clarity on the patient’s priorities, decision makers need guidance as to what types of treatment decisions are well-served by advance care planning and which are not.

Planning for future care in frailty is challenging because the nature and timing of health crises are unpredictable, and therefore the context in which decisions arise often differs from what patients imagine. Although many advanced directive frameworks encourage making decisions about specific treatments long before they are needed, such anticipatory choices are often oversimplified and may lead individuals to commit to interventions that do not align with their future functional state, or goals. A more practical approach is to distinguish between interventions that can be appropriately decided in advance and those that should remain open to thoughtful reconsideration in real time. We offer the following Red–Green framework as an anecdotally effective clinical approach to support advance care planning in frailty. In this framework, future interventions fall into one of two categories:

- **The Red category**: The Red category includes treatments that are unacceptable to the patient based on cultural or religious considerations (e.g., blood transfusions when contraindicated by religious or cultural beliefs) or that clinicians would not recommend because they are unlikely to improve survival or quality of life (e.g., tube feeding in severe stage dementia; hemodialysis in end stage heart failure).
- **The Green category**: The Green category includes all other treatments, including those the individual may prefer to avoid based on their current state of health. Treatments in the Green category require thoughtful, contextual decision making at the time they are needed because their risks, benefits, and alignment with patient values depend heavily on the context of the health crisis, the degree of frailty, comorbidities, and the anticipated outcomes.

Only treatments in the Red category are appropriate for advanced decisions. It may seem counterintuitive to avoid advance decisions for treatments in the Green category, but such far-in-advance choices are prone to affective forecasting errors, where individuals overestimate the burden of disability and underestimate their ability to adapt and cope [49,50]. This can lead patients to decline life sustaining treatment that may well offer an acceptable quality of life. Instead of focusing on specific treatment decisions in the green category, advance care planning conversations and advanced directives should focus on values, priorities, and desired outcomes, with the SDM participating as a witness and partner in these discussions. Cognitive ability is also critical to the capacity to adapt physically and mentally to new disability. When cognitive impairment, such as dementia, is present, it may be difficult for individuals to recall restrictions, implement adaptations, or appreciate the value of external supports or reframe expectations in ways that sustain mental wellbeing. Despite being inappropriate for advanced decisions, treatments in the Green category are still guided by patient values.

Many healthy individuals may have few or no treatments in the Red category. As frailty progresses, treatments move from the Green category into the Red category—reflecting a widening scope of interventions that become medically inappropriate in the present state of health and therefore suitable for advance decision making. The Red Green framework clarifies which decisions should be made in advance and which should remain open to contextual judgment, supporting more practical and value-aligned care planning in frailty. It preserves the complexity of frailty, supports autonomy, and provides a practical structure for advance care planning that helps substitute decision makers apply the patient’s values in the moment when it matters most.

**Case application.** The clinician introduces the idea of which treatments are appropriate for decision making at this time (Red-category) by underscoring that interventions such as CPR, treatments requiring Intensive Care Unit (ICU) admission or intubation are unlikely to provide meaningful benefit given Mr. L’s dementia stage and moderate frailty. Mrs. L agrees that these interventions would not help restore him to a quality of life aligned with his values. The clinician affirms that other, less invasive interventions such as hospitalization for symptom control, anti-anginal medications, and future treatment of infections—each require contextual judgment based on patient preferences as articulated by Mr. L’s wife (Green-category).

### 2.7. Task 7: Use a Structured Approach to Just-in-Time Decision Making

As described above, in frailty, an acute health crisis is an expected event. The nature, timing and context of the health crisis means that dignity and personhood are best served by just-in-time decision making with the best available understanding about the patient’s current quality of life, conditions and prognosis. This approach builds on the concepts of the Frailty Cycle (Task 2) and achieving a current shared understanding of health (Task 4) to help the decision maker navigate the immediate health crisis by considering the nature of the crisis; the health trajectory in the weeks and months preceding the crisis; the available treatment options; and their implications for dignity, symptom burden, function and progression of other health conditions. Together, these elements inform a treatment plan that aligns with the patient’s values and prognosis. Under ideal conditions, the patient is able to confer with the provider who is already familiar with these elements, but in a specialized health system where continuity is scarce, providing the decision maker (the patient or SDM) and their decisional understudy with the right questions to ask of the provider during the crisis can help inform a thoughtful discussion about the options [51]. These questions could include:

1. **Which health conditions are easily treatable and which are not?**
  This question helps decision makers understand that some treatments may effectively target a specific condition without altering the progression of other, more serious illnesses. It encourages realistic expectations about what treatment can accomplish within the broader context of frailty.
2. **How will frailty impact treatment risk?**
  This question prompts clinicians to consider how frailty influences treatment tolerance and functional recovery—not just survival. For example, hip arthroplasty may repair a fracture in someone with severe dementia, but the risks to cognition are significant, and advanced dementia greatly limits the likelihood of withstanding post-operative recovery and regaining independent mobility.
3. **Will the proposed treatment require time in hospital?**
  Time in hospital can be associated with suffering and negative impacts on personhood and dignity. When life expectancy is limited, strategies that reduce hospital exposure—such as home-based rehabilitation or enhanced community supports—may better align with the patient’s priorities and protect quality of life.
4. **How can symptoms be safely and effectively managed?**
  This question underscores that, in the setting of multiple life-limiting conditions, symptom control may best meet the patient’s values and goals. It opens the door for conversations about what palliative care can provide. For example, treating pneumonia-related distress may be possible without hospitalization or antibiotics if comfort is the priority. It also reinforces the importance of avoiding interventions for asymptomatic conditions (e.g., elective investigation of an incidental lung nodule), which may pose more harm than benefit in frailty.
5. **If the decline in overall health cannot be reversed, what can we do to promote comfort and dignity in the time left?**
  This question invites reflection on whether a shift toward comfort-focused care may now be most appropriate. It frames a palliative approach to care as an active strategy that supports quality of life and preserves personhood, rather than as a withdrawal of care.

**Case application.** Eighteen months later, Mr. L presents to the Emergency Department after experiencing a fall and hip fracture. In the Emergency Department, he is febrile, disoriented and has not been able to swallow his medications or fluids. A chest x-ray shows changes suggestive of an aspiration pneumonia. The clinician reviews the prior discussions that have taken place with Mrs. L and gathers further information from her to understand his current frailty. Mrs. L describes the functional changes that have occurred since the NSTEMI including the need for hands-on assistance with each step of bathing and dressing due to changes in cognition. This function is consistent with severe stage dementia. The clinician reviews the operative and non-operative treatment options for hip fracture in the setting of severe stage dementia and coronary artery disease including the implications of coronary artery disease for operative risk and the implications of severe stage dementia and delirium for participation in post-operative physiotherapy, as well as the implications of dysphagia for nutrition and recovery. The clinician and Mrs. L collaboratively identify a non-operative plan to address pain and to focus on comfort. Palliative care is initiated, and Mr. L dies 3 days later in hospital.

## 3. Conclusions

Frailty-informed decision making is an advanced clinical skill—one that requires structure, intentionality, and practice. As with any medical procedure, there is a defined purpose (aligning care with the patient’s values and prognosis) and an obligation to minimize harm, including the psychological burden placed on patients and substitute decision makers. The seven tasks described in this framework function like procedural steps: each must be performed with competence before advancing to the next. Competency builds through repetition and increasing levels of complexity, ultimately enabling clinicians to navigate the uncertainty and urgency inherent in acute health crises. By approaching communication and care planning with the same discipline used in technical procedures, clinicians can reliably support autonomy, preserve personhood, and reduce low-value and harmful interventions for people living with frailty.

This framework is based on clinical experience, and existing evidence and has been validated in clinical practice [52]. Guidance may require adaptation across different healthcare settings, legal contexts, and models of substitute decision making. The applicability of certain recommendations may vary in settings with limited geriatric expertise, restricted access to collateral information, or variable continuity of care. Further research should explore feasibility, effectiveness, and system-level enablers to support consistent use in routine practice.

## Data Availability

No new data were created or analyzed in this study.
